# Supplementary material for: Deletion of wfs1 Impairs Oligodendrocyte Precursor Cells Dorsal Distribution and Myelination Through the wfs1-hmgcs1 Axis in Zebrafish
Source: Biology (Basel). 2026 Aug 16;15(16):1402. doi: 10.3390/biology15161402 (PMC13510151; doi:10.3390/biology15161402)
Supplement: Supplementary file 1 [file biology-15-01402-s001.zip › File S1-Transcriptome sequencing data and differentially expressed genes (DEGs) in wfs1-- zebrafish at 5 dpf/2.KEGG/wfs1KOvsWT/ALL/src/dre00620.html]

dre00620


- dre:406702

- Up regulated genes

406702(2.99135335361027)

- dre:406473

- Up regulated genes

406473(3.55746093515464)

- dre:445233

- Up regulated genes

445233(5.61580861179048)

- dre:335715

- Up regulated genes

335715(3.34138689148967)

- dre:100005587

- Down regulated genes

100005587\_1(-1.54424530950344)
- dre:100536795

- Down regulated genes

100536795(-1.64304185838866)
- dre:368239

- Up regulated genes

368239(1.14034759711527)
- dre:100006238

- Down regulated genes

100006238\_1(-1.31531604695891)

- dre:559403

- Up regulated genes

559403\_1(1.4336766768898)

- dre:406702

- Up regulated genes

406702(2.99135335361027)

- dre:445326

- Up regulated genes

445326\_1(2.09813524726464)

- Down regulated genes

445326(-1.62876055255802)

- dre:116517

- Up regulated genes

116517(5.15873267159645)
- dre:393427

- Down regulated genes

393427(-1.25663853074503)

Close
